# Clinical and laboratorial aspects of hens egg allergy in childhood

**DOI:** 10.1186/1939-4551-8-S1-A85

**Published:** 2015-04-08

**Authors:** Eliana Toledo

**Affiliations:** 1 Faculty of Medicine of São José Do Rio Preto, Brazil

## Background

The prevalence of hen´s egg allergy is estimated in 0.5 to 2.5 per cent of young children. In view of this, clinical and laboratory aspects of hen´s egg allergy were assessed in children at a specialized clinic.

## Methods

Prospective study conducted from 1997 to 2014 in children at the age of 0 to 18 years old with hen´s egg allergy. Eosinophilia was considered over 400 cells / mm ³. The technique used for specific IgE was immunofluorescence. The result of prick test was the average of the papule, considered positive papules over 3mm. The results were presented as mean values.

## Results

It was evaluated 159 children, with 58.5% male. The mean age at diagnosis was 1 year and 11 months old. Among the patients followed 25.1% achieved oral tolerance, and this was achieved after 1 year and 10 months of diagnosis. The clinical manifestations evaluated were: atopic eczema (27.0%), urticaria (24.5%), atopic eczema associated with urticaria (13.2%), anaphylaxis (8.8%), gastrointestinal tract manifestations (8.8%), respiratory tract manifestations (8.8 %), urticaria and angioedema (7.5%) and angioedema (1.8%). The following laboratory aspects evaluated were: Eosinophilia was present in 30.1% of patients. The mean total IgE was 1108.8 IU/ml. The specific IgE to hen´s egg was 9.3 IU/ml and to the allergenic component ovomucoide was 1.9 IU/ml. The mean wheal to hen´s egg in prick test was 4.8mm.

## Conclusions

It was concluded that allergy to hen´s egg was more common in males. The opportune diagnosis of allergy to hens egg was considered late, on the other hand, oral tolerance was achieved rapidly. The most common clinical manifestation was atopic eczema and mean total IgE was elevated.

